# Identification of a Novel Antibiotic from Myxobacterium *Stigmatella Eracta *WXNXJ-B and Evaluation of its Antitumor Effects *In-vitro*

**Published:** 2014

**Authors:** Dahong Wang, Jiangfeng Yuan, Wenyi Tao

**Affiliations:** a *College of Food and Bioengineering, Henan University of Science and Technology. *; b *College of Food and Bioengineering, Henan University of Science and Technology,Luoyang, Henan, China. *; c *School of Biotechnology and Key Laboratory of Industrial Biotechnology, Ministry of Education, Jiangnan University, Wuxi, Jiangsu, China.*

**Keywords:** Quinoxalone, Myxobacterium* Stigmatella eracta* WXNXJ-B, Structure identification, Antitumor bioactivity

## Abstract

This work was to isolate and identify the bioactive secondary metabolite which was produced by myxobacterium *Stigmatella eracta *WXNXJ-B, and to evaluate its antitumor and apoptosis-inducing effects. The results showed that one novel compound (molecular formula C_29_H_25_NO_3_) was isolated, purified by Sephadex LH-20 column chromatography and preparative RP-HPLC, and identified as 5-(6-benzyl-quinolin-3-ylmethyl)-6- phenyl-3,7-dioxa- bicycle [4.1.0] heptan-3-one (named as quinoxalone) according to its UV, IR, HRMS and NMR spectra. The compound showed strong antitumor activity on B16, HepG2, MCF-7, SGC-7901, MDA-MB231 and CT-26 six tumor cell lines* in-vitro*. Nevertheless, it showed less cytotoxic to the mouse normal spleen cells (IC_50_ was 836.27 ± 13.02 µg mL^-1^). The cytotoxic study on HepG2 cells *in-vitro* showed that quinoxalone could induce the change of cell nuclear and arrested the cell division in the S and G2/M phase. Our results suggest that quinoxalone could be a potential anti-cancer agent.

## Introduction

Myxobacteria are gram-negative unicellular rod shaped bacteria with suitable culture pH between 5.0 and 8.0. They can be frequently isolated from soil, dung of herbivorous animals and other decaying organic material (28, 29). They are common but unusual bacteria characterized by gliding behavior and forming fruiting body, and not obtained by the routine method used in culturing bacteria due to their complicated life cycle (1). They can produce a wide variety of secondary metabolites which often show high pharmacological or anti-fungicidal activity, such as quinoids, alkaloids and polyenic compounds, *etc* (26). The first myxobacterial antibiotic, ambruticin, was isolated from *Polyangium cellulosum* in 1977 (4). Then, the structure of myxothiazol was firstly reported by Gerth* et al. *(10). With the past thirty years, myxobacteria had increasingly gained attention as producers of natural products with biological activity (11). Ixabepilone, one epothilone derived from *Sorangium cellulosum*, shows good water solubility and can be produced by fermentation (16,19). So, it was approved in 2007 by the FDA for use in the treatment of aggressive metastatic or advanced breast cancer (6,12). Due to their extraordinary ability to produce novel classes of secondary metabolites, myxobacteria represent a very promising source for the discovery of new lead structures and novel natural products (10, 32). 

In continuing effort to find novel bioactive metabolites from myxobacteria, the researchers in our lab obtained five myxobacteria which showed strong antitumor bioactivity *in-vitro *(14), and they isolated and identified a novel antitumor metabolite, phoxalone, from the fermentation broth of *S.cellulosum* WXNXJ-C (13). In addition, we had reported that the metabolites from *S.eracta *WXNXJ-B showed high antitumor bioactivity *in-vitro*, but the bioactive substance was not isolated and identified (31). The objective of this study is to isolate, identify the antitumor bioactive substance from the fermentation broth of *S.eracta *WXNXJ-B, evaluate its bioactivity on different cell lines, and explore apoptosis-inducing effects on HepG2 tumor cell line* in-vitro*.

## Experimental

*Microorganism and cell lines*

The strain myxobacteria *Stigmatella eracta* WXNXJ-B was used throughout the study. HepG2 human liver carcinoma cell line, B16 mouse melanoma cell line, CT-26 murine colon carcinoma cell line MDA-MB231 and MCF-7 human breast cancer cell line, SGC-7901 gastric carcinoma tumor cell line and mouse spleen cells were provided by college of Medicine and Pharmaceutics, Jiangnan University, China. All cells were cultured in RPMI-1640 medium (Gibco, USA) with 10% inactivated fetal bovine serum (Gibco, USA), streptomycin (100 µg mL^-1^) and penicillin (100 U mL^-1^) at 37 ^o^C in a 5% CO_2_ incubator. Epothilone B and Paclitaxel were purchased from Sigma-Aldrich Co.

*Culture conditions*

Medium for slant was CY medium as described by Guo *et al.* (2007). Medium composition for seed and fermentation cultures was (per litre) 10 g potato starch, 8 g glucose, 2 g defatted milk powder, 2 g yeast extract, 8 mg EDTA, 1 g CaCl_2_, 1 g MgSO_4_**•**7H_2_O. In the fermentation medium, about 20 g l^-1 ^XAD-16 adsorbent resins (Rohm and Haas, USA) were added to adsorb the bioactive metabolites. The pH was adjusted to 7.2 by the addition of 1 mol l^-1^ HCl or 1 mol l^-1^ NaOH. *S. eracta *WXNXJ-B was grown on CY medium at 30 ^o^C for 5 days, then inoculated in seed medium for flask culture at 30 ^o^C with shaking at 150 rev min^-1^. After 2 days, the seed broth was transferred to fermentation medium and fermented at 30 ^o^C with shaking at 150 rev min^-1 ^for 7 days.

*Isolation and* *purification of* *quinoxalone*

At the end of the fermentation, the XAD-16 adsorbent resins were separated by sieving and rinsed with water to remove cells and fermentation broth. The resins were extracted with methanol at room temperature for 12 h. The extract was concentrated at 45 ^o^C and further purified by partition between water and chloroform. The chloroform extract was concentrated at 45 ^o^C and followed by chromatography using a Sephadex LH-20 column (10 μm ×12 mm ×400 mm, with a gradient of 20-100% (v/v) methanol at flow rate 1.5 mL min^-1^). The fraction eluted with 90% methanol was purified using a preparative RP-HPLC (Waters 510, USA) with a C_18_ column (Sephax, USA, 5.0 μm ×10 mm×150 mm, with mobile phase 80% (v/v) methanol at flow rate 3 mL min^-1^). The antitumor activities of different fractions were evaluated with HepG2 tumor cell line using MTT method (33). Quinoxalone which showed the strongest antitumor bioactivity was obtained. 

*Structural elucidation of* *quinoxalone*

UV spectrum was recorded on an Ubest-50 UV/VIS spectrophotometer (Jasco, Japan). FT-IR spectrum in KBr was recorded on a Nicolet Nexus 470 infrared spectrometer (Thermo, USA). The high-resolution MS (HRMS) was obtained by MALDI-Q-TOF (Waters, USA) in the positive ESI mode and the spectral data were processed by MassLynx 4.1 software (Waters). The ^1^H- NMR was detected with 600 MHz Varian Inova (Varian, USA) in CD_3_SOCD_3_ solution. The ^13^C- NMR was detected with 400 MHz Varian Inova (Varian) in CD_3_SOCD_3_ solution.

*Evaluation of quinoxalone* *in-vitro (MTT assay)*

B16, CT-26, HepG2, DMA-MB231, MCF-7, SGC-7901 and mouse spleen cells were used to evaluate the antitumor effects of quinoxalone. Cells were harvested, counted, diluted and seeded into 96-well plates at a density of approximately 7000 cells per well. After incubating for 24 h, two hundred microlitre of the medium with different concentration quinoxalone which was dissolved into dimethylsulfoxide (DMSO) was added into per well. To avoid the influence of DMSO, medium containing 0.5% DMSO was used as a control. Incubation was carried out for another 48 h. The cell viability was assessed by MTT (colorimetric 3-[4, 5--2-Yl]-2, 5-diphenyl tetrazolium bromide) assay. Twenty microlitre of MTT solution (5 mg mL^-1^) was added into each well and incubated at 37 ^o^C for additional 4 h. The formazan product was dissolved by adding 200 μL DMSO and shaked for 5 min. Then, the absorption was measured at 570 nm with a Multiskan MK3 microplate reader (Labsystems, Finland).The inhabitation rate was calculated as follows: inhabitation rate= (1-OD_ treated_/OD_ negative control_)×100%. Data were obtained from six repeat experiments.

*Cell cycle analysis*

The DNA and the proportion of HepG2 cells treated with quinoxalone in different phases were analyzed with flow cytometer (5, 20). HepG2 cells which were treated with different concentration quinoxalone were harvested by centrifugation, and then fixed with cold 75% ethanol for 24 h. The cells were washed with phosphate buffered saline (PBS), and then stained with PI solution (10 mg mL^-1 ^RNase A, 50 mg ML^-1 ^PI, and 0.1% Triton X-100). After incubated for 1 h at 4 °C in the dark, cells were measured in the FACScan flow cytometer, and the data were analyzed using Cellfit Analysis Software. 

*Fluorescence microscop* *e* *observation of HepG2 cells*

To observe the change in nuclear structure, HepG2 cells were plated onto glass cover slips in 6-well plates and treated with 0 and 5 µg mL^-1^ quinoxalone for 48 h. Then, cells were washed twice with PBS, fixed with 1% glutaraldehyde, stained with Hoechst 33342 (Sigma, USA) for 15 min at room temperature (25). Nuclear morphology was examined by fluorescence microscope (Olympus, Tokyo, Japan).

*Statistical analysis*

Data were represented as mean ± SD. Statistical differences were determined by Student’s t-test. Samples with p-values of p < 0.05 were considered statistically different.

## Results

*Structure identification of quinoxalone*

**Figure 1:**
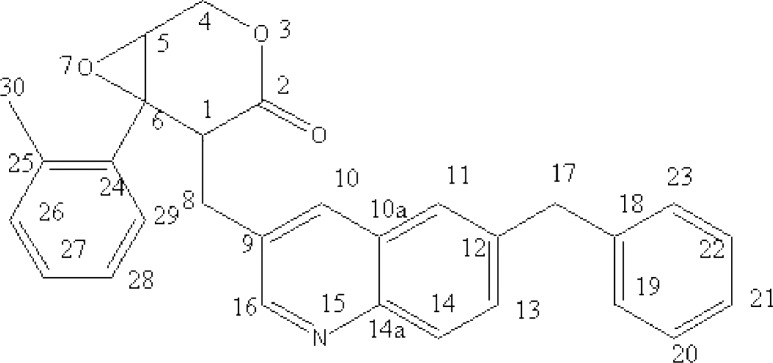
The chemical structure of quinoxalone

After isolation and purification, a brownish-red compound was obtained. The molecular formula of the compound was determined to be C_29_H_25_NO_3_ by combined UV, IR, HRMS and NMR spectrometry. The major physical and analytic properties of the compound were summarized as below: UV (in methanol) λ_max _253 and 339 nm; IR (KBr) 3050, 2958, 2927, 1730, 1639, 1534, 1454, 1395, 1235, 1103, 789, 742, 701 cm^–1^; ^1^H-NMR (CD_3_SOCD_3_, 600 MHz) δ: 8.15 ppm (1H, s, quinolione), 8.12 ppm (^1^H, d, quinolione), 7.85 ppm (1H, d, quinolione) , 7.49 ppm (1H, s, quinolione),7.47 ppm (1H, s, quinolione), 7.35 ppm (2H, t, phenyl), 7.26 ppm (2H, d, phenyl), 7.18 ppm (3H, t, phenyl), 6.99 ppm (1H, d, phenyl),6.77ppm (1H, t, phenyl), 4.36 ppm (2H, d, O-CH_2_-CH), 4.06ppm (2H, s,C-CH_2_- C), 3.26 ppm (1H, t, O-CH-CH_2_-O), 3.23 ppm (1H, t, CH_2_-CH-C), 2.96 ppm (2H,d, -CH_2_-), 2.35 ppm (3H,s,C-CH_3_); 1D ^13^C-NMR (CD_3_SO CD_3_, 400 MHz) δ: 60.29 (C-1),68.95 (C-2), 172.85 (C-4), 50.56 (C-5), 64.64 (C-6), 29.57(C-8), 129.93 (C-9), 132.24 (C-10),126.66(C-10a), 124.65 (C-11), 135.59 (C-12), 131.25(C-13), 126.66(C-14), 145.08(C-14a),150.57 (C-16), 41.54(C-17), 143.65(C-18),129.10 (C-19),129.10 (C-20), 127.22(C-21), 127.93(C-22), 128.54 (C-23), 139.55 (C-24), 136.33 (C-25), 150.10 (C-26), 126.24(C-27), 125.32 (C-28), 127.93(C-29), 16.22 (C-30) (Fig.S5); HREIMS m/z 436.1758 (calcd for C_29_H_25_NO_3_, 435.1864). The structure of compound was depicted by analyzing above spectrum data (Figure 1). According to the physical and analytic properties, the new compound was [1R, 6R]-5- (6-benzyl- quinolin-3-ylmethyl)-6- phenyl-3, 7-dioxa-bicycle [4.1.0] heptan-4-one and it was named as quinoxalone. 

According to the handbook of organic chemical analysis and spectrum, the characteristic peak at 253 nm and 339 nm indicated phenyl group and lactone ring, respectively. The HREIMS spectrum of quinoxalone showed protonated molecular ion [M+H]^+ ^at m/z 436.1942, while the calculated molecular weight was 436.1738 Da. The HREIMS spectrum revealed that the molecular weight of the compound was 435.1864 and the molecular formula C_29_H_25_NO_3_. The IR spectrum showed absorptions for phenyl ring (1534, 1454, 789, 742, 701 cm^-1^), quinoline ring (1639 cm^-1^) and lactone (1730 cm^-1^). The ^13^C-NMR spectrum showed 29 signals. The complete structure of quinoxalone was elucidated by ^1^H-NMR and ^13^C-NMR experiments. 

*Antitumor evaluation of quinoxalone on* *cell lines in-vitro*

B16, CT-26, HepG2, DMA-MB231, MCF-7 and SGC-7901 tumor cell lines were used to evaluate the antitumor effects of quinoxalone* in-vitro*. As we can see from Table 1, quinoxalone showed strong antitumor bioactivity to the above six cell lines after treating the cells with it at various time. The IC_50_ values indicated that quinoxalone has the higher inhibition ability to CT-26 (IC_50 _2.12 ± 0.19 µg mL^-1^) and B16 (IC_50_ 2.23 ± 0.14 µg mL^-1^) for 48 h, followed by HepG2 (IC_50_ 2.41±0.32 µg mL^-1^) and DMA-MB231 (IC_50 _3.08 ± 0.21 µg mL^-1^). It showed lower cytotoxicity to SGC7901 and MCF-7 cell lines. Compared with the reported antitumor drugs, Paclitaxel and Epothilone B, the antitumor bioactivities of quinoxalone on MCF-7, B16 and HepG2 cell lines were less than those of Epothilone B and Paclitaxel* in-vitro*. The influence on the growth of mouse spleen cells by quinoxalone was checked. The proliferation of mouse normal spleen cells was slightly influenced at the low dose. The growth of cells treated with the higher concentration was significantly inhibited and IC_50_ was 836.27 ± 13.02 µg mL^-1^. So, quinoxalone showed relative safety to the mouse normal spleen cells. The results indicated that quinoxalone could selectively kill the cells and induced tumor cell to die in all probability by apoptosis manner.

**Table 1:** Evaluation of bioactivity of quinoxalone to different cell lines* in-vitro*

**Cell line**	**IC**_50_ **(** **μ** **g mL** ^-1^ **)**			**IC**_50_ ** (** **μ** **g mL** ^-1^ **) at 48 h**	
	24 h	48 h	72 h	Paclitaxel	Epothilone B
B16	5.48±0.26	2.23±0.14	2.02±0.12	1.62±0.15*	0.73±0.1
CT-26	8.33±0.38*	2.12±0.19	2.04±0.23*		
MCF-7	10.12±0.5*	6.05±0.25*	5.83±0.28	2.94±0.17*	1.32±0.12
SGC7901	9.29±0.37**	5.72±0.32	5.65±0.24**		
HepG2	13.8±0.45	2.41±0.32*	2.09±0.35	2.03±0.18	0.45±0.11*
DMA-MB231	7.58±0.41**	3.08±0.21	2.93±0.18		
mouse spleen cells	>1000	836.27±13.02	763.41±15.2**		

Paclitaxel and Epothilone B were the positive controls. Each value represents mean ± standard deviation of six separate experiments. Significant differences with control were designated as

* P < 0.05 and **P < 0.01.

**Figure 2:**
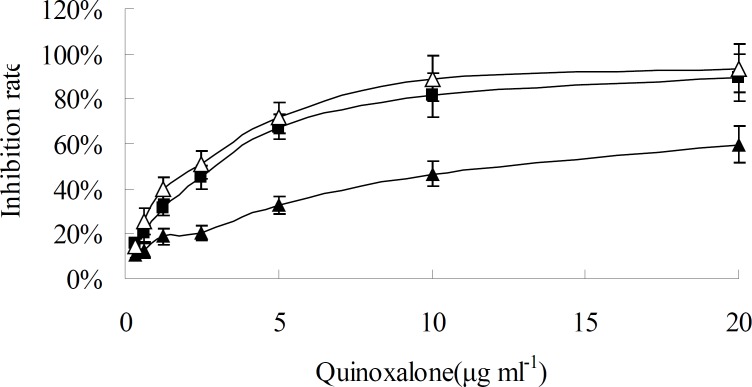
Time and concentration effect of quinoxalone on HepG2 cells* in-vitro*. HepG2 cells were treated with different concentration quinoxalone (0.32, 0.625, 1.25, 2.5, 5.0, 10.0 and 20.0 µg mL^-1^) and different time (24, 48 and 72 h). Black triangle, black square and white triangle were 24, 48 and 72 h, respectively. Data represent the mean ± SD of six independent experiments

**Figure 3:**
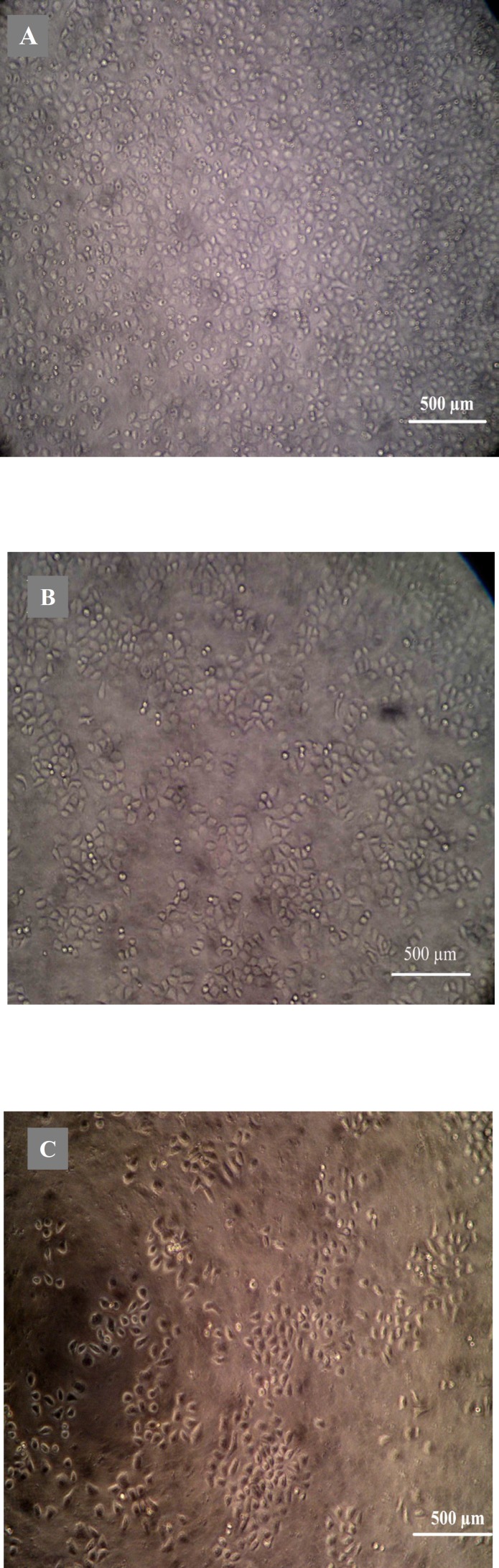
The influence of different concentration quinoxalone to HepG2 cells observed by inverted microscope for 48 h (magnification 100).

When HepG2 cells were treated with various concentration of quinoxalone and various treating time, the growth of cells was significantly inhibited at dose- and time-dependent manner, as shown in Figure 2 and Figure 3. When the concentration was under 10 µg mL^-1^, the change of inhibition rate was obvious as the concentration increase of quinoxalone. Compared to the control, significant decrease of the amount of HepG2 cells treated with quinoxalone (5 and 10 µg mL^-1^) for 48 h was observed. Furthermore, the cell treated for 48 h began to have poorly adherence to the culture flask. The change of inhibition rate was slight as the concentration increase of quinoxalone when the dose was above 10 µg mL^-1^. The IC_50_ on HepG2 cells was 13.8 ± 0.45 µg mL^-1^ after treating for 24 h, but the IC_50_ were 2.41 ± 0.32 and 2.09 ± 0.35 µg mL^-1 ^after treating for 48 h and 72 h, respectively. The treating effects for 48 h and 72 h were similar. So, the results suggested that the best treating time was 48 h. 

A: 0 µg mL^-1^; B: 5 µg mL^-1^; C: 10 µg mL^-1^

**Figure 4:**
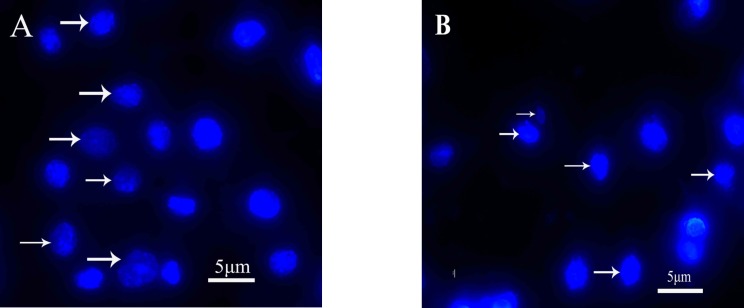
Fluorescence micrographs of HepG2 cells stained with Hoechst 33342 (magnification 400). Cells were treated with 0 μmg mL^-1^ (A) and 5 μg mL^-1^ (B) quinoxalone for 48 h. White arrow were the normal cells in Figure A. White arrow were the apoptosis cells in Figure B

Morphological analysis of cell character observed by inverted microscope showed that HepG2 cells treated with 5 µg mL^-1^ quinoxalone started to change their shape (they shrunk and started to round up) and the total amount of living cells was distinctly less than those of the control. Moreover, large number of dead cells was suspended in the culture medium. With higher concentration of quinoxalone, cell shrinkage and blebbing on cell membrane could be observed by scanning electron microscope. These alterations were even more expressed following 24 h treatment (data not shown). At the same time (48 h treatment), we also noticed changes in nuclear morphology on HepG2 cells under a fluorescence microscope (Figure 4). The results showed that the cells had nuclear shrinkage and condensed chromatin of nucleus (Figure 4B). 

*Effect of quinoxalone on cell cycle of HepG2 cells*

**Figure 5:**
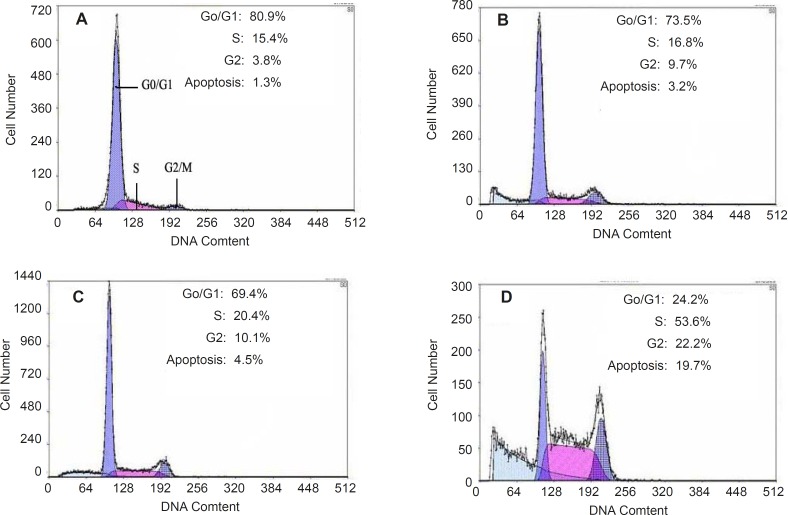
Cell cycle analysis of HepG2 cell line treated with quinoxalone at different concentration of (A) 0 µg mL^-l^, (B) 2.5 µg mL^-l^, (C) 5 µg mL^-l^, (D) 10 µg mL^-l ^for 48 h. Results are presented as the number of cells versus the amount of DNA as indicated by fluorescence intensity

Apoptosis of HepG2 cells could be induced by quinoxalone using the PI staining method. After HepG2 cells treated with quinoxalone (at dose of 0, 2.5, 5 and 10 µg mL^-1^) for 48 h, the total amount of cells in the subsequent cell cycle phases differed from those of the control. Figure 5 showed that 80.9% cells treated without quinoxalone were in the G0/G1 phase, but 24.2% cells treated with 10 µg mL^-1 ^quinoxalone were in the G0/G1 phase. But, compared with the control, the total amount of cells treated with 10 µg mL^-1 ^quinoxalone in S phase significantly elevated from 15.4% to 53.6% and from 3.8% to 22.2% in G2/M phase. Moreover, the number of cells in S and G2/M phase increased with a dose-dependent manner. Because the most tumor cells were accumulated in S phase and in G2/M phase, HepG2 cells could not be duplicated, and finally led to die. 

## Discussion

Microbial metabolites are the most important chemotherapeutic agents for the cancer. They appeared around 1940 with the discovery of actinomycin and since then many anticancer compounds have been isolated from microorganisms (6). Myxobacteria have become known as multi-producers of natural bioactive products. In the past years, more than 100 new core structures plus approximately 500 derivatives have been described. Furthermore, some secondary metabolites are rarely produced by other sources (13). Up to now, epothilone has been used in the clinic, and many other metabolites have currently been evaluated in preclinical studies (32). Moreover, most substances derived or isolated from myxobacteria exhibited the direct action on the proliferation of the tumor cells at certain concentrations. Ambruticin, Epothilones, Spirodienal C and Phoxalone had been revealed to show the inhibition ability on the tumor cells proliferation by inducing the cell apoptosis. The ability to induce cell apoptosis is an important property of the candidate anti-cancer drugs (9).

Our previous results indicated that the metabolites from myxobacterium *S.eracta* WXNXJ-B showed strong bioactivity on some tumor cell lines* in-vitro* by MTT method, but the bioactive substance and mechanism was unclearly (31). In the present study, we showed a novel antitumor compound which was isolated from the culture broth of *S. eracta *WXNXJ-B, was able to significantly inhibit the growth of some tumor cell lines The compound, C_29_H_25_NO_3_, is identified according to UV, IR, HRMS and NMR spectra, and named as quinoxalone (Figure 1). It is one of quinoline alkaloids and a novel compound according to the Novelty Assessment Report provided by Station of Science and Technology of Education of Minister, East China University of Science and Technology, China. Quinolines are widely distributed in nature and constitute an important class of natural compounds. They are produced by plants, bacteria and fungi. Many quinolines show the bioactive of antitumor, antibacteria, antimalaria and antifungi (21, 22). For example, it was reported the compound, 2-heptyl-4-hydroxyquinoline N-oxide, which was isolated from the culture of Pseudomonas aeruginosa, could inhibited the growth of Staphylococcus aureus and other Gram-positive organisms, and also played an important role in signaling transfer. (15, 24). Zhang *et al.* reported that marine-derived fungus *Aspergillus sydowi *PFW-13 produced an indole- quinazolines alkaloid which showed strong antitumor bioactivity (34). 

Cancer is a disease characterized by uncontrolled cellular growth and apoptosis obstacle (2, 7). The inhibition of tumor cellular proliferation and induction of apoptosis are efficient methods for tumor therapy (20). So, induction of apoptosis in tumor cells is the target for many candidate antitumor drugs (25). Lin et al reported that the lichenin from *Cladonia* *furcata* exhibited inhibition on HL-60 or K562 cells in concentration-dependent manner between 200 and 800 mg l^-1^ for 4-6 days (23). It was similar to our results, in this work we evaluated the cytotoxicity of quinoxalone *in-vitro* using the method of microculture MTT-tetrazolium. Our data demonstrated that quinoxalone exhibited significantly cytotoxic effects towards B16, HepG2, MCF-7, SGC-7901, MDA-MB231 and CT-26 tumor cell lines (Table 1). The time course studies on HepG2 cells showed that quinoxalone at different concentrations for 24 h had slight inhibitory effect on the cell proliferation, whereas, at the same concentrations, the cell proliferation obviously decreased for 48 h and 72 h (Figure 2). Moreover, the cell viability gradually decreased with the concentration increasing at same time course (Figure 3). So, the results showed that quinoxalone was able to significantly inhibit cells proliferation with a time- and dose-dependent manner. In addition, quinoxalone have slight influence on normal mouse spleen cells at the same concentration applied on HepG2 tumor cells. The results suggested that the quinoxalone selectively killed tumor cells and normal cells. It can be concluded that quinoxalone killed tumor probably by apoptosis. 

Apoptosis is a regulated process characterized by cell shrinkage, nuclear disintegration, selective degradation of DNA, and formation of apoptotic bodies with a relatively intact plasma membrane (30). Morphological changes in the cell shape and chromatin condensation are basic and the oldest criteria for identification of apoptotic cells (18). These changes are caused by activation of proteolytic enzymes, caspases, the central executioners of apoptosis (8). In this study, HepG2 cells treated with different concentration of quinoxalone showed the characteristics of apoptosis induction compared with the control, for example, apoptotic bodies, nuclear shrinkage and condensed chromatin (Figure 4). The result confirmed that quinoxalone was able to induce cell apoptosis in HepG2 cells. 

Mammalian cells have evolved a complex defence network to maintain genomic integrity by inhibiting the fixation of permanent damage. Cell-cycle check.

points prevent cells with damaged genomes from undergoing DNA replication or mitosis (17,27).Targeting cell cycle is an attractive approach for treatment of cancer. Many anticancer agents have been found to arrest cell cycle (19). For example, Paclitaxel induced G2/M phase arrest in human Saos-2 cells (3). Guo *et al.* reported that Phoxalone suppresses H446 cell line proliferation with cell cycle arrest in the G2/M phase (14). In this work, to determine if quinoxalone influences the cell cycle of HepG2 cells, we examined cell cycle phase distribution of the treated cells by flow cytometry. Our data clearly showed that in quinoxalone-treated HepG2 cells, the significant increase of S phase cells was accompanied by a decrease of G0/G1 phase cells and a little change of G2/M phase cells (Figure 5). Thus, the blockage effect of quinoxalone occurred at the G1/S transitions, and the increase of cell numbers in S phase was clearly due to the decrease of cells in the G0/G1 phase. These results clearly suggested that quinoxalone affected cell proliferation by arresting cell cycle progression and inducing apoptosis. Furthermore, the apoptosis induced by quinoxalone was mediated via arresting cell cycle in S phase; that is to say, quinoxalone could induce apoptosis in a cell cycle-dependent manner. However, the detailed mechanism of this process has not yet been resolved. So, the further research on the molecular mechanisms of quinoxalone effecting on the cells’ cycle is necessary.

In conclusion, we showed that a novel alkaloid was isolation and purified from the fermentation broth of myxobacterium *S.eracta* WXNXJ-B. Its chemical structure was elucidated and also named as quinoxalone. This is the first report of this compound. It had a significant cytotoxic effect to tumor cells *in-vitro *and could induce apoptosis in HepG2 cells in a time and dose dependent manner. It mainly arrested the cell division in the S and G2/M phase by flow cytometery. But, the comprehensive mechanism of apoptosis needs further research in the future. This makes quinoxalone interesting for further investigations as a potential anti-cancer drug.

## References

[1] Ahn J, Li X, Zee O, Soraphinol B (2007). a new acyloin compound produced by Sorangium cellulosum. Bull Korean Chem. Soc..

[2] Bartek J, Lukas C, Lukas J Checking on DNA damage in S phase. Nat Rev. Mol. Cell Bio..

[3] Bruna P, Lorenza B, Marco T, Valeria M, Gerry M, Antonio G (1999). Paclitaxel induces apoptosis in Saos-2 cells with CD95L upregulation and Bcl-2 phosphorylation. Exp Cell Res..

[4] Connor D, Greenough R, Von Strandtmann M (1977). W-7783, a unique antifungal antibiotic. J Org. Chem..

[5] Cui F, Li Y, Xu Y, Liu Z, Huang D, Zhang Z, Tao W (2007). Induction of apoptosis in SGC-7901 cells by polysaccharide-peptide GFPS1b from the cultured mycelia of Grifola frondosa GF9801. Toxicol Vitro.

[6] Demain A, Sanchez S (2009). Microbial drug discovery: 80 years of progress. J Antibiot..

[7] Ding L, Liu B, Qi L, Zhou Q, Hou Q, Li J, Zhang Q (2009). Anti- proliferation, cell cycle arrest and apoptosis induced by a natural xanthone from Gentianopsis paludosa Ma, in human promyelocytic leukemia cell line HL-60 cells. Toxicol Vitro.

[8] Earnshaw WC, Martins LM, Kaufmann SH (1999). Mammalian caspases: structure, activation, substrates, and functions during apoptosis. Ann Rev. Biochem..

[9] Frankfurt OS, Krishan A (2003). Apoptosis-based drug screening and detection of selective toxicity to cancer cells. Anti-Cancer Drugs.

[10] Gerth K, Irschik H, Reichenbach H (1980). Myxothiazol, an antibiotic from Myxococcus fulvus (Myxobacterales) I. Cultivation, isolation, physico- chemical and biological properties. J. Antibiot..

[11] Gerth K, Pradella S, Perlova O, Beyer S, Müller R (2003). Myxobacteria: proficient producers of novel natural products with various biological activities-past and future biotechnological aspects with the focus on the genus Sorangium. J Biotechnol..

[12] Goodin S, Kane MP, Rubin EH (2004). Epothilones: mechanism of action and biologic activity. J Clin. Oncol..

[13] Guo W, Tao W, Xu Z, Ao Z (2007). Directed-screening of myxobacteria producing high bioactive antitumor metabolites. Nat Prod. Res. Dev..

[14] Guo W, Tao W (2008). Phoxalone, a novel macrolide from Sorangium cellulosum: structure identification and its antitumor bioactivity in-vitro. Biotechnol Lett..

[15] Hodgkinson J, Bowden S, Galloway Wa Spring D, Welch M (2010). Structure-activity analysis of the Pseudomonas quinolone signal molecule. J Bacteriol..

[16] Hofle G, Reichenbach H, Cragg GM, Kingston DG, Newman DJ (2005). Epothilone, a myxobacterial metabolite with promising antitumor activity. Anticancer agents from natural products.

[17] Jakopec S, Dubravcic K, Polanc S, Kosmrlj J, Osmak M (2006). Diazene JK-279 induces apoptotis-like cell death in human cervical carcinoma cells. Toxicol Vitro.

[18] Kerr JF, Wyllie AH, Currie AR (1972). Apoptosis: a basic biological phenomenon with wide-ranging implications in tissue kinetics. British J Cancer.

[19] Kim RH, Peters M, Jang Y, Shi W, Pintilie M, Fletcher GC, DeLuca C, Liepa J, Zhou1 Lily, Snow B (2005). Binari CR, Manoukian AS, Bray MR, Liu FF, Tsao MS and Mak TW. DJ-1, a novel regulator of the tumor suppressor PTEN. Cancer cell.

[20] Kinloch RA, Treherne MJ, Furness ML, Hajimohamadreza I (1999). The pharmacology of apoptosis. Trends Pharm Sci..

[21] Kitagawa W, Tamura T (2008). A quinoline antibiotic from rhodococcus erythropolis JCM 6824. J Antibiot..

[22] Kunze B, Hofle G, Reichenbach H (1987). The aurachins,new quinoline antibiotics from myxobacteria: production, physico-chemical and biological properties. J Antibiot..

[23] Lin X, Cai Y, Li Z, Chen Q, Liu Z, Wang R (2003). Structure determination, apoptosis induction, and telomerase inhibition of CFP-2, a novel lichenin from Cladonia furcata. Biochim Biophys. Acta.

[24] Machan ZA, Taylor GW, Pitt TL, Cole PJ, Wilson R (1992). 2-heptyl-4- hydroxylquinoline N-oxide, an antistaphilococcal agent produced by Pseudomonas aeruginosa. Antimicrob Agents Chemother..

[25] Peng B, Chang Q, Wang L, Hu Q, Wang Y, Tang J, Liu X (2008). Suppression of human ovarian SKOV-3 cancer cell growth by Duchesnea phenolic fraction is associated with cell cycle arrest and apoptosis. Gynecol Oncol..

[26] Reichenbach H, Hofle G (1993). Biologically active secondary metabolites from myxobacteria. Biotechnol Adv..

[27] Shackelford RE, Kaufmann WK, Paules RS (1999). Cell cycle control, checkpoint mechanisms, and genotoxic stress. Environ Health Perspect..

[28] Shimekets LJ, Dworkin M, Reichenbach H, Balows A, Trüper T, Dworkin M (2006). The myxobacteria. prokaryotes.

[29] Velicer GJ, Vos M (2009). Sociobiology of the myxobacteria. Annu Rev. Microbiol..

[30] Vrba J, Doležel P, Vičar J, Ulrichová J (2009). Cytotoxic activity of sanguinarine and dihydrosanguinarine in human promyelocytic leukemia HL-60 cells. Toxicol Vitro..

[31] Wang D, Tao W (2010). Nutrient regulation of bacterial growth and production of antitumor metabolites in Stigmatella WXNXJ-B fermentation. World J Microbiol.Biotechnol..

[32] Wenzel S, Muller R (2009). Myxobacteria-‘microbial factories’for the production of bioactive secondary metabolites. Mol Biosyst..

[33] Yamaguchi H, Paranawithana SR, Lee ME, Huang Z, Bhalla KN, Wang H (2002). Epothilone B analogue (BMS-247550) mediated cytotoxicity through induction of bax conformational change in human breast cancer cells. Cancer Res.

[34] Zhang M, Fang Y, Zhu T, Zhao W, Gu Q, Han X, Zhu W (2007). Study on indole-quinazolines alkaloids from marine-derived fungus Aspergillus sydowi PFW-13 and the anti-tumor activities. Chin Pharm..

